# Resistin in Urine and Breast Milk: Relation to Type of Feeding and Anthropometry at 1-Month

**DOI:** 10.3390/pediatric14010013

**Published:** 2022-02-14

**Authors:** Irena Santosa, Hiromichi Shoji, Kentaro Awata, Yoshiteru Arai, Hiroki Suganuma, Toshiaki Shimizu

**Affiliations:** 1 Department of Pediatrics and Adolescent Medicine, Graduate School of Medicine, Juntendo University, Tokyo 113-8421, Japan; s-irena@juntendo.ac.jp (I.S.); k-awata@juntendo.ac.jp (K.A.); yo-arai@juntendo.ac.jp (Y.A.); tshimizu@juntendo.ac.jp (T.S.); 2 Department of Pediatrics, Faculty of Medicine, Juntendo University, Tokyo 113-8421, Japan; hsuganu@juntendo.ac.jp

**Keywords:** resistin, leptin, urine, breast milk, term infants

## Abstract

Breast milk contains adipokines such as resistin and leptin and is known for its protective effect against obesity and insulin resistance. This pilot study aims to evaluate the correlation between resistin levels, feeding types (breast milk and formula), and anthropometric parameters in healthy 1-month-old term infants. Urine and breast milk samples were collected from 32 infants and their mothers at 1 month postpartum. Twelve infants were included in the breastfed group, while thirteen infants comprised the breastfed-dominant mix-fed group, and seven infants the formula-dominant mix-fed group. Using ELISA kits, we analyzed resistin levels in the infants’ urine and the mothers’ breast milk, and leptin levels in breast milk. Urinary resistin levels among the three groups were not significantly different. There was no correlation between the following: urinary resistin levels in the breastfed group with resistin levels in breast milk; resistin levels in urine with infant’s body weight and weight gain; resistin levels in breast milk with weight, age, and BMI of mothers and leptin levels in breast milk. This study suggests that the type of feeding does not affect resistin levels in term infants and resistin level does not affect growth in early infancy.

## 1. Introduction

Breastfeeding is recommended as the optimal source of nutrition for infants, to support their growth and development as well as long-term health [1]. Several studies indicate that breastfeeding has a protective effect against excess weight gain and insulin resistance in childhood [2,3]. The protective role of breast milk can be attributed not only to its nutritional composition such as proteins, but also bioactive substances that have been recently investigated. Bioactive substances such as adipokines are involved in the development of physiological functions [4].

Resistin was first discovered in 2001 and it is an adipocyte-derived secretory factor. Along with leptin and adiponectin, resistin belongs to the group of adipocytokines [5]. Resistin is a low molecular weight protein, weighing 12.5 kDa, consisting of 94 amino acids, encoded by the RETN gene located on chromosome 19p13.3 [6]. In humans, resistin is mainly secreted by macrophages in the adipose tissue [7]. Resistin is also found in large amounts in other tissue, such as bone marrow, lungs, circulating mononuclear cells, endothelial cells, vascular smooth muscles, and human placenta [8]. Resistin antagonizes the action of insulin and affects insulin sensitivity, with higher levels inconsistently observed among obese than in lean adults [9]. Resistin has been suggested to have an important role in obesity, insulin resistance, and diabetes [10].

Leptin, which also belongs to the adipocytokine group, is a protein with a molecular weight of 16 kDa, consisting of 167 amino acids, and is encoded by the obesity (ob) gene localized on chromosome 7q31.3 in humans [11]. Leptin is mainly synthesized by the white adipose tissue, in proportion to the amount of body fat mass. It is also produced in the human placenta and plays a potential role in fetal growth [12]. In addition, it is also produced in the mammary glands, where it is secreted by the epithelial cells in fat globules of breast milk [13].

Although there is some information about resistin and leptin levels during the neonatal period [14,15], previous studies have not assessed the urinary resistin levels of infants. Therefore, in this study we investigated the levels of resistin in the urine and breast milk in the early postnatal period.

The aim of this study is to evaluate the influence of infants’ feeding method and its correlation with the anthropometric parameters of infants. Some studies show that breastfeeding has a protective effect against excess weight gain and obesity in childhood [2,3]. Obesity is characterized by excess accumulation of lipids in the adipose tissue. The levels of resistin, an adipocyte-secreted hormone, are increased in obese adults. Resistin is associated with insulin resistance and an increased risk of type 2 diabetes. Based on these facts, we hypothesized that breast-fed infants would have a lower level of resistin than formula-fed infants.

## 2. Materials and Methods

We conducted a pilot study for one year between September 2018 and August 2019. Term infants who were appropriate for gestational age, from single pregnancies with no perinatal complications including asphyxia, infections, or bleeding were included in this study. This study was carried out by the opt-out method of the hospital website. All subjects gave their informed consent and the protocol for this study was reviewed and approved by the Institutional Review Board of Juntendo University Hospital (19–183).

A total of 32 infants were included in this retrospective study. We divided the subjects into three groups according to the methods of feeding. Twelve infants in the breast-fed (BF) group did not receive formula milk during the study period, 13 infants in the breast milk dominant mix-fed group (B > F) received less than 100 mL/kg of formula milk per day. The remaining 7 infants included in formula milk dominant mix-fed group (B < F) received more than 100 mL/kg of formula milk per day.

Formula milk was given alone or in combination with breast milk only when the breast milk was inadequate, solely based on the mother’s decision. The amount of intake of formula milk was recorded daily by the mothers for 7 days before the one-month check-up day.

At the regular one-month health check-ups, the same physician performed the interviews and medical examinations. The infants in this study did not show any signs of infection, such as temperature instability or respiratory distress. Body weight measured on this day was used to count the body weight gain by subtracting it with the birth weight and was divided by the days from birth to one-month check-up day.

We collected spot urine samples from infants in a urine bag and breast milk samples from the infants’ mothers who attended regular one-month health check-ups. Breast milk samples were centrifuged twice at 1800× *g* for 30 min to eliminate the fat layer. Then both urine and breast milk samples were stored at −80 °C until use.

We analyzed urinary and breast milk resistin levels (Human Resistin ELISA kit; Arigo biolaboratories, Hsinchu City, Taiwan) and breastmilk leptin (Human Leptin Quantikine ELISA Kit; R&D Systems, Minneapolis, MI, USA) in infants using an enzyme-linked immunosorbent assay kit. We also measured the absorbance of urinary creatinine by enzymatic methods using a JCA-BM8060 automatic analyzer (Beckman Coulter, Fullerton, CA, USA) at 545 nm. The values of urinary resistin are shown as ng/mg creatinine.

The results are presented as mean ± standard deviation (SD). We used the Wilcoxon rank-sum test to compare categorical variables between the groups. A *p*-value of <0.05 was considered statistically significant. All statistical analyses were conducted using JMP statistical software, version 12.0 (SAS Institute Inc., Cary, NC, USA).

## 3. Results

The clinical characteristics and anthropometric indices of infants and their mothers included in this study are shown in Table 1. There were no significant differences in the age and anthropometric indices of mothers and infants among the three groups (Table 1). The mean gestational age and weight of 12 infants (7 male) in the BF group were 39.5 weeks and 3.02 kg, 39.7 weeks, and 3.04 kg in the 13 infants (9 male) from the MF (B > F) group and 39.5 weeks and 2.99 kg in the 17 infants (4 male) from the MF (F > B) group.

**Figure 1:**
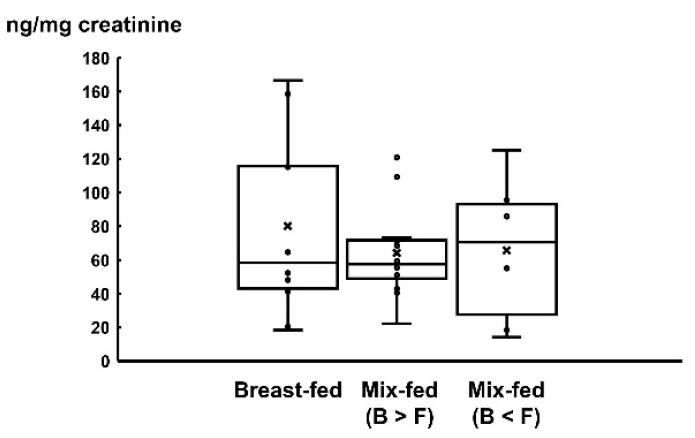
Urinary resistin level in breast-fed group, breast milk dominant mix-fed group, and formula milk dominant mix-fed group.

**Figure 2:**
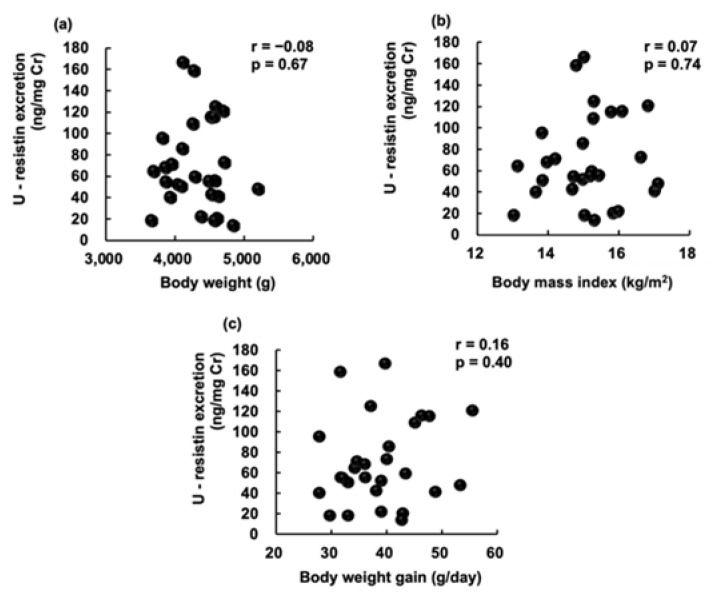
Correlation of urinary (U) resistin level to (**a**) infant’s body weight; (**b**) infant’s body mass index (BMI); (**c**) body weight gain. Each dot represents an independent sample.

Urinary resistin levels in the three groups were not significantly different (BF group: 80.2 ± 54.8 ng/mg creatinine, MF (B > F) group: 64.2 ± 27.8 ng/mg creatinine, and MF (B < F) group: 65.7 ± 44.3 ng/mg creatinine) (Figure 1). We found no correlation between urinary resistin levels with body weight, weight gain at 1 month, and BMI (Figure 2).

Urinary resistin concentrations were measured by ELISA and expressed as box plot; the central rectangle spans the first quartile to the third quartile. A segment inside the rectangle shows the median and ‘whiskers’ above and below the box show the locations of the minimum and maximum.

**Figure 3:**
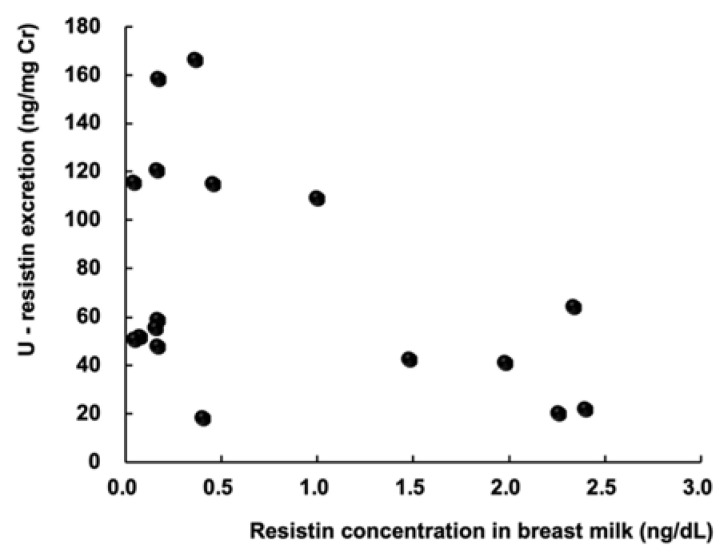
Association between urinary resistin level in breast-fed infants and resistin concentration in the mother’s breast milk.

**Figure 4:**
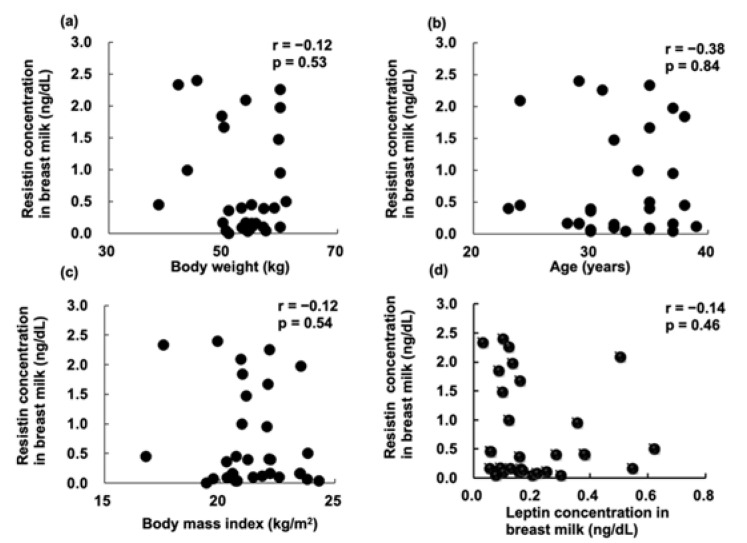
Correlation between resistin concentration in the breast milk and (**a**) body weight; (**b**) age; (**c**) body mass index (BMI); and (**d**) leptin concentration in mothers’ breast milk at 1 month post-partum.

In this study, we measured resistin levels in both urine and breast milk. There was no correlation between urinary resistin levels in the BF group and the resistin levels in breast milk (r: −0.12, *p*: 0.53) (Figure 3). There was also no correlation between resistin levels in breast milk and the mother’s weight, age, BMI, and leptin levels in breast milk (Figure 4).

## 4. Discussion

To the best of our knowledge, this was the first study that examined the relationship between the method of feeding and urinary resistin levels in early infancy. In 2008, resistin was identified in the breast milk [15,16]. Some studies reported that resistin is produced and secreted by the mammary epithelial cells. These studies also showed that resistin is transferred from the mother’s blood into the breast milk [17]. In one study, high levels of resistin (about 1.71 ng/mL) were observed in the colostrum [16]; however, the mean resistin levels found in breast milk in this study was 0.72 ng/mL. It is known that resistin levels in breast milk decrease significantly and in parallel to the serum resistin levels of mothers. The decrease of serum resistin in mothers might be due to the loss of fat stores in breastfeeding women.

Yamamoto et al. [18] found that urinary resistin, insulin, and leptin concentrations were significantly correlated with blood concentrations. They stated that glomerular filtration membranes are permeable at a certain rate for proteins up to 20–30 kDa. It has been confirmed by the gel filtration method that insulin, leptin, resistin, and GH leaks into urine with an intact molecular weight. Although, Kopf et al. reported that high molecular weight (HMW) proteins with a size > 250 kDa would not pass the filtration barrier.

Savino et al. [15] reported that the mean resistin level in breast milk was 0.18 ng/mL and did not demonstrate any correlation with anthropometric parameters of infants at 3 months of age. They also reported a positive correlation between the level of resistin in breast milk and that in the serum of breast-fed-infants. They hypothesized that consumption of breast milk contributes to serum resistin levels of the infant [15]. However, in this study, we found no significant correlation between the level of resistin in breast milk and urinary resistin levels in breast-fed infants. Some infants with low intake of breast milk showed high urinary resistin levels. Endogenous resistin production might play a role in the regulation of metabolic homeostasis considering its metabolic function.

In this study, we found no correlation between resistin levels and leptin levels in the breast milk at 1 month. One study found a positive correlation between serum resistin and leptin in infancy [15]; other studies also reported this correlation in cord blood on the third day after birth [19,20].

Schuster et al. [21] demonstrated that leptin levels increase in breast milk during the first month postpartum. A higher concentration of circulating leptin was found in breastfed infants [22]. Although it is known that the adipokines, resistin, and leptin might inhibit the formation of adipose tissue [23,24], further research is necessary to define its protective effect against obesity and insulin resistance in breastfed infants.

This study had some limitations. The sample size was small and the influence on renal development was not evaluated. We could not perform serum resistin and leptin measurement because we selected a non-invasive approach to assess resistin and leptin in healthy term infants. Hutcheson et al. previously reported that serum resistin, but not urinary resistin, was correlated with markers of renal dysfunction in an adult patient with lupus nephritis [25].

## 5. Conclusions

Our study suggests that the method of feeding does not correlate with urinary resistin level in healthy term infants, and urinary resistin level does not correlate with growth in early infancy. Non-invasive urinary analysis using ELISA might be useful for assessing resistin level in early infancy instead of serum resistin level.

## Figures and Tables

**Table 1:** General characteristics of the subjects.

	Breast-Fed 12 (7/5)	Mix-Fed (B > F) 13 (9/4)	Mix-Fed (B < F) 7 (4/3)
Mothers			
Age (years)	32.1 ± 4.5	33.2 ± 4.1	32.9 ± 5.8
Body height (cm)	158.6 ± 4.7	157.0 ± 6.5	160.5 ± 4.4
Body weight before pregnancy (kg)	50.3 ± 5.0	47.9 ± 5.7	52.0 ± 4.9
BMI before pregnancy (kg/m^2^)	20.0 ± 1.6	19.4 ± 1.4	20.2 ± 1.6
Body weight at delivery (kg)	61.6 ± 5.4	59.2 ± 7.2	63.5 ± 3.7
Body weight at one month (kg)	54.0 ± 5.1	52.0 ± 6.3	56.1 ± 3.5
BMI at one month (kg/m^2^)	21.5 ± 1.5	21.2 ± 2.0	21.2 ± 2.0
Infants (at birth)			
Gestational age (week)	39.5 ± 1.10	39.7 ± 1.2	39.5 ± 1.1
Body weight (g)	3022.2 ± 255.8	3042.9 ± 306.7	2993.4 ± 239.2
Body weight z-score	0.1 ± 0.7	0.1 ± 0.8	0.0 ± 0.6
Body length (cm)	48.3 ± 1.8	48.0 ± 1.5	48.4 ± 1.2
Body length z-score	−0.4 ± 0.8	−0.6 ± 0.8	−0.4 ± 0.7
Infants (at one month)			
Age (days)	33.8 ± 5.2	33.8 ± 4.0	31.7 ± 2.2
Body weight (g)	4355.0 ± 432.3	4364.5 ± 359.2	4097.7 ± 441.1
Body length (cm)	53.4 ± 1.3	53.4 ± 1.0	53.2 ± 1.7
Body weight gain (g/day)	39.1 ± 8.9	39.2 ± 7.6	34.5 ± 5.6
Body weight gain (%)	44.7 ± 16.7	44.0 ± 11.5	36.8 ± 7.0
Intake volume of formula (mL/kg/day)	0.0 ± 0.0 *^,#^	25.5 ± 24.1 *	130.7 ± 30.4

* *p* < 0.01 compared with the mix-fed (B < F) group. ^#^ *p* < 0.01 compared with the mix-fed (B > F) group. Data are presented as means ± SD.
